# Protective Effect of miR-204 on Doxorubicin-Induced Cardiomyocyte Injury via HMGB1

**DOI:** 10.1155/2020/8819771

**Published:** 2020-11-19

**Authors:** Youyou Du, Guanghui Liu, Luosha Zhao, Rui Yao

**Affiliations:** Department of Cardiology, The First Affiliated Hospital of Zhengzhou University, Zhengzhou, China

## Abstract

The toxicity of doxorubicin (DOX) limits its clinical application. Nevertheless, at present, there is no effective drug to prevent DOX-induced cardiac injury. miR-204 is a newly discovered miRNA with many protective effects on cardiovascular diseases. However, little research has been done on the effects of miR-204 on DOX-induced cardiac injury. Our study is aimed at investigating the effect of miR-204 on DOX-induced myocardial injury. An adenoassociated virus system was used to achieve cardiac-specific overexpression of miR-204. Two weeks later, the mice were intraperitoneally injected with DOX (15 mg/kg) to induce cardiac injury. H9c2 myocardial cells were used to validate the role of miR-204 in vitro. Our study showed that miR-204 expression was decreased in DOX-treated hearts. miR-204 overexpression improved cardiac function and alleviated cardiac inflammation, apoptosis, and autophagy induced by DOX. In addition, our results showed that miR-204 prevented DOX-induced injury in cardiomyocytes by directly decreasing HMGB1 expression. Moreover, the overexpression of HMGB1 could offset the protective effects of miR-204 against DOX-induced cardiac injury. In summary, our study showed that miR-204 protected against DOX-induced cardiac injury via the inhibition of HMGB1, and increasing miR-204 expression may be a new treatment option for patients with DOX-induced cardiac injury.

## 1. Introduction

Doxorubicin is currently one of the most effective and widely used anticancer drugs, but its specific cardiotoxicity seriously hinders its clinical application [1]. Doxorubicin-induced cardiotoxicity often leads to irreversible degenerative cardiomyopathy and congestive heart failure, which are associated with high morbidity and mortality [2, 3].

Doxorubicin-induced cardiotoxicity involves a variety of mechanisms, including cardiomyocyte apoptosis and autophagy [4, 5]. However, although many studies have described the potential mechanism of doxorubicin-induced cardiotoxicity, there is still a lack of effective cardioprotective methods available. In view of its efficacy and wide application in cancer, it is important to develop new therapeutic strategies to alleviate cardiotoxicity and dysfunction caused by doxorubicin.

Recently, some studies have focused on the involvement of microRNAs (miRNAs) in the study of DOX-induced toxicity [6]. miRNAs are small noncoding RNAs that regulate gene expression at the posttranscriptional level by regulating their target mRNAs [7]. There is increasing evidence that miRNAs play key roles in different physiological/pathological processes. More importantly, there is growing evidence that miRNAs are involved in all cardiac functions, including myocardial apoptosis, autophagy, and electrical signal conductance [8]. Moreover, clinical and animal studies have shown that miRNAs are potential regulators of DOX-induced cardiotoxicity [9]. One specific miRNA, miR-204, was originally thought to be a tumor suppressor in human cancer [10]. miR-204 has been reported to play a role in regulating apoptosis, inflammation, oncogenesis, and the development of immune cells. More recently, research has shown that miR-204 plays an important role in many cardiovascular diseases, such as hypertrophic cardiomyopathy and myocardial ischemia-reperfusion injury [11]. However, the functional and molecular mechanisms of miR-204 in protecting against DOX-induced cardiac injury have not been reported.

In our study, we investigated the potential functions of miR-204 in DOX-induced cardiac injury. The findings of our study showed that the expression of miR-204 was decreased in DOX-treated hearts. Moreover, we found that overexpression of miR-204 could attenuate the adverse effects caused by DOX. We further found that miR-204 reduced cell inflammation, apoptosis, and autophagy by directly decreasing HMGB1 expression.

## 2. Materials and Methods

### 2.1. Animal Experimental Design

All procedures were performed according to the National Institutes of Health Guide for the Care and Use of Laboratory Animals and approved by the Animal Care and Use Committee of the First Affiliated Hospital of Zhengzhou University. The Chinese Academy of Medical Sciences Institute of Laboratory Animal Science (Beijing, China) provided male C57B/L6J mice. The mice were divided into 4 groups: the GFP-NS group, AAV-miR-204-NS group, GFP-DOX group, and AAV-miR-204-DOX group (*n* = 12 in each group). A single administration of DOX (15 mg/kg intraperitoneal injection) was used to establish a short-term cardiac injury model. An equal volume of saline was administered to the control mice [12]. The mice were given adenoassociated virus encoding murine miR-204 (AAV-miR-204) and the control vector (AAV-green fluorescent protein [GFP]) by injection to overexpress miR-204 in vivo. Briefly, 14 days before DOX injection, the mice were administered the AAV vectors (2 × 1011 genome copies/mouse, a total 50 *μ*L per mouse) by injection into the retroorbital venous plexus as described in a previous study [13] .

### 2.2. Recombinant Adenoassociated Virus (AAV) 9 Construct

AAV9-GFP and AAV9-miR-204 were constructed and generated by Vigene Biosciences (Shandong, China). Briefly, the murine miR-204 gene was cloned into the p-ENTER vector by the AsiS I and Mlu I restriction sites. The p-ENTER plasmid containing the desired gene and AAV vector pAV-C-GFP were cotransfected into 293T cells to obtain the pAAV-MCS plasmid. Then, the recombinant plasmid pAAV-MCS was transfected into AAV-293T cells. Three days after transfection, AAV9 vector-producing 293T cells were harvested for vector purification. Real-time PCR was used to quantify the AAV viral particles.

### 2.3. Echocardiography and Hemodynamics

Echocardiography and hemodynamic measurements were performed as described in our previous study [14]. Briefly, a MyLab 30CV ultrasound (Esaote SpA, Genoa, Italy) was used with a 10 MHz linear array ultrasound transducer to measure echocardiographic parameters. A Millar catheter transducer (SPR-839; Millar Instruments, Houston, TX) and PVAN data analysis software were used to measure hemodynamic parameters.

### 2.4. Biochemical Determination

Forty-eight hours after DOX injection, mouse blood was collected and centrifuged to collect the serum. The Fuji DRI-CHEM chemical analyzer (Fujifilm, Tokyo, Japan) was used to measure cardiac troponin I (cTnI), creatine kinase isoenzymes (CK-MB), and lactate dehydrogenase (LDH).

### 2.5. Histological Analysis, Immunohistochemistry, and TUNEL Staining

Hematoxylin-eosin (H&E) and picrosirius red staining and immunohistochemistry were performed as described in our previous study [15].

The LV myocyte area was calculated from 100-200 cells per group. Primary antibodies, including anti-4-hydroxynonenal (4-HNE; 1 : 100), anti-CD45 (1 : 100), anti-CD68 (1 : 100), and anti-TNF-*α* (1 : 100), were used for immunohistochemical analysis. A commercial kit (Millipore, Billerica, MA) was used to stain TUNEL-positive cells.

### 2.6. Cell Culture and Treatment

The Shanghai Institutes for Biological Sciences (Shanghai, China) provided the H9c2 cells. The cells were cultured in DMEM (C11995; Gibco, Grand Island, NY, USA) supplemented with 10% FBS (FBS, 10099; Gibco), penicillin (100 U/mL), and streptomycin (100 mg/mL) (15140; Gibco) in a humidified incubator (SANYO 18M, Osaka, Japan) with 5% CO_2_ at 37°C. Cells were transfected with adenoassociated viral vectors containing the entire rat HMGB1 cDNA coding region to overexpress HMGB1. Cells were transfected with adenoassociated viral vector encoding GFP as controls. Before DOX treatment, the cells were transfected with miR-204 mimics (50 nM) or negative controls (NC mimic) (RiboBio, Guangzhou, China). Cells were treated with DOX (1 *μ*mol/L) [16] for 24 h to induce cell injury in vitro.

### 2.7. ELISA and Lactate Dehydrogenase (LDH) Measurement

TNF-*α*, IL-1, and IL-6 were quantified by using commercial ELISA kits (Technology Co., Ltd., Boster Biological, China). LDH levels were measured using commercial kit reagents (Biotein, Shanghai, China) according to the manufacturer's instructions.

### 2.8. Evaluation of LC3 Puncta

The cells were washed with phosphate-buffered saline (PBS), fixed with RCL2® (RCL2-CS24L; ALPHELYS, Plaisir, France), and permeabilized in 0.1% Triton™ X-100 in PBS. The cells were then incubated with anti-LC3 (Cell Signaling Technology, Boston, MA, USA) at a dilution of 1 : 100 in 1% goat serum. The cells were then incubated with Alexa Fluor 488-conjugated goat anti-mouse immunoglobulin (Ig)G (A11001; Invitrogen Life Technologies, Carlsbad, CA, USA) secondary antibodies. The cells were then incubated with SlowFade Gold Antifade reagent with DAPI (S36939; Invitrogen Life Technologies). An AX60 objective (BX51 Microscope, Olympus) was used to observe the images.

### 2.9. Real-Time Polymerase Chain Reaction and Western Blotting

Total RNA was isolated and purified as described in our previous study [17, 18]. Total RNA was extracted from frozen mouse cardiac tissue or cardiomyocytes using a TRIzol reagent (15596-026; Invitrogen). RNA (2 *μ*g of each sample) was reverse-transcribed into cDNA using oligo (DT) primers and a Transcriptor First Strand cDNA Synthesis Kit (04896866001; Roche). The LightCycler 480 SYBR Green Master Mix system (Roche) was used for quantification. The GAPDH gene was used as a reference.

Total proteins were extracted as described in our previous study [15]. An ice-cold radioimmunoprecipitation assay buffer (containing 50 mM Tris–HCl, 150 mM NaCl, 1% Triton X-100, 1% sodium deoxycholate, and 0.1% SDS) was used to extract proteins from cardiomyocytes and heart tissue. A 10% sodium dodecyl sulfate polyacrylamide gel was used to separate the proteins. The following primary antibodies were used: Bax, Bcl-2, cytochrome c, HMGB1, ATG5, ATG12, LC3, p62, and glyceraldehyde 3-phosphate dehydrogenase (GAPDH) (1 : 1000 dilution, Cell Signaling Technology, Boston, MA, USA). Horseradish peroxidase-conjugated secondary antibodies were used (Jackson ImmunoResearch Laboratories, West Grove, PA, USA). Image Lab 5.2.1 software was used for quantification. The GAPDH protein was used as a reference.

### 2.10. Luciferase Reporter Assay

To predict the potential binding sites of miR-204 and HMGB1, miRanda software was used (http://www.microrna.org/microrna/getGeneForm.do). The pRL-CMV luciferase reporter plasmid was constructed by cloning the 3′-untranslated region (3′-UTR) of the human HMGB1 gene containing the potential miR-204 binding sites. The mutated control was cloned with a mutated sequence. Then, 293T cells were transfected with the indicated constructs. The Dual-Luciferase Reporter Assay System (Promega, Madison, WI) was used to measure the firefly and Renilla luciferase activities.

### 2.11. Statistical Analysis

All data are expressed as the mean ± SEM. One-way analysis of variance (ANOVA) followed by a post hoc Tukey's test was used to compare differences between groups. Differences between two groups were analyzed by an unpaired, two-sided Student's *t* test. A *P* value less than 0.05 was considered statistically significant.

## 3. Results

### 3.1. Doxorubicin Decreased miR-204 Levels In Vivo and In Vitro

**Figure 1:**
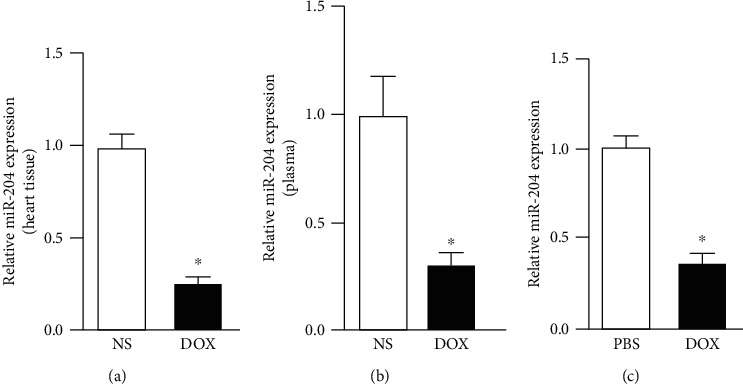
miR-204 was downregulated in doxorubicin- (DOX-) induced cardiac injury. (a) miR-204 levels in the heart tissue of mice 8 days after the injection of DOX (*n* = 6). *P* < 0.05 versus NS. (b) miR-204 levels in the plasma of mice 8 days after the injection of DOX (*n* = 6). *P* < 0.05 versus NS. (c) Real-time PCR was used to measure the expression of miR-204 in DOX-treated cardiomyocytes (*n* = 6). *P* < 0.05 versus PBS.

To investigate whether miR-204 is associated with DOX-induced cardiac injury, real-time PCR was used to measure the level of miR-204. The level of miR-204 in the hearts of mice treated with doxorubicin decreased significantly (Figure 1(a)). In addition, miR-204 was also reduced in the plasma of mice treated with DOX (Figure 1(b)). The in vitro results showed that doxorubicin exposure could significantly reduce the expression of miR-204 in H9c2 cells (Figure 1(c)).

### 3.2. Overexpression of miR-204 Alleviated DOX-Induced Inflammatory Responses and Apoptosis in Cardiomyocytes

**Figure 2:**
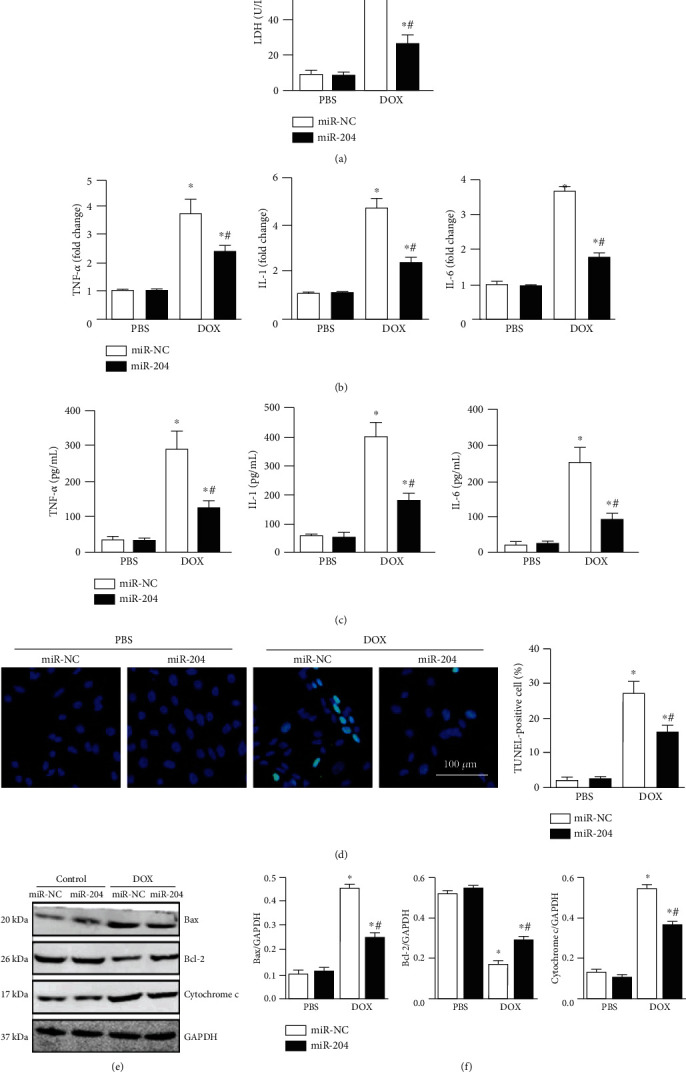
miR-204 inhibited DOX-induced inflammation and cell loss. (a) Cardiomyocyte viability in the indicated groups (*n* = 6). (b) Measurement of inflammation-related genes by real-time PCR (*n* = 6). (c) Measurement of inflammatory factors by ELISA (*n* = 6). (d) Representative images and the quantitative analysis of the TUNEL assay (*n* = 4). (e, f) Western blot and quantitative analysis in each group (*n* = 6). ^∗^*P* < 0.05 versus PBS+miR-NC and ^#^*P* < 0.05 versus DOX+miR-NC.

Then, we examined whether miR-204 could affect DOX-induced cardiac injury. miR-204 overexpression was achieved by transfecting H9c2 cells with agomir-miR-204 (Figure 2(a)). We first determined the impact of miR-204 on DOX-impaired cardiomyocyte viability, and our results showed that miR-204 overexpression could block the inhibitory effect of DOX on cell viability. Proinflammatory cytokine expression was increased in DOX-induced cardiomyocytes [17], whereas miR-204 overexpression blocked the increased proinflammatory cytokine expression (Figures 2(b) and 2(c)). Previous studies confirmed that apoptosis is markedly increased in DOX-induced cardiomyocytes [17, 18]. We then examined the severity of apoptosis and evaluated the potential signaling pathways related to apoptosis in cardiomyocytes. Our results showed that overexpressing miR-204 markedly decreased the proportion of apoptotic cells (Figure 2(d)). The expression of Bax and cleaved cytochrome c was upregulated in the DOX-treated hearts compared with control hearts. Conversely, the expression of Bcl-2 was prominently lower in DOX-induced hearts than in control hearts. However, overexpressing miR-204 significantly attenuated the increased expression of Bax and cytochrome c and abrogated the decrease in Bcl-2 expression in DOX-treated hearts (Figures 2(e) and 2(f)). These results show that overexpressing miR-204 attenuates DOX-induced cardiomyocyte inflammation and apoptosis.

### 3.3. miR-204 Overexpression Improved Heart Function in DOX-Induced Mice

**Figure 3:**
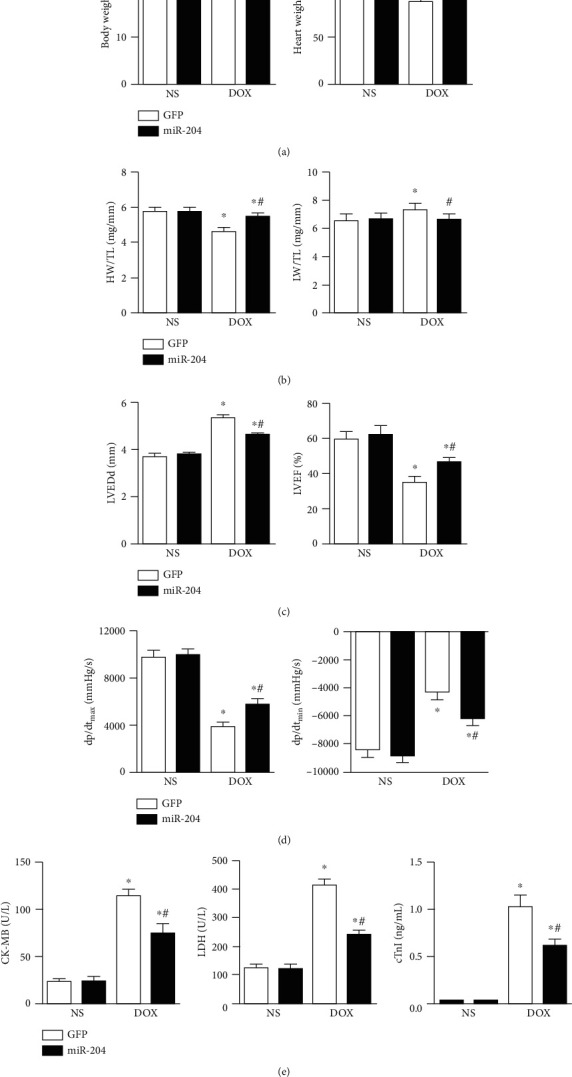
miR-204 improved cardiac function in DOX-treated mice. (a) Body weight and heart weight in each group (*n* = 9‐12). (b) The ratio of the heart weight (HW)/tibia length (TL) and lung weight (LW)/TL (*n* = 9‐12). (c) Left ventricular end-diastolic diameter (LVEDd) and ejection fraction (EF) of mice in each group (*n* = 9‐12). (d) Hemodynamic analysis of mice in the indicated groups (*n* = 8‐11). (e) The levels of serum biochemical parameters (CK-MB, LDH, and cTnI) in each group (*n* = 6). ^∗^*P* < 0.05 versus NS+GFP and ^#^*P* < 0.05 versus DOX+GFP.

To study the role of miR-204 in cardiac injury induced by DOX, miR-204 overexpression in mouse hearts was achieved by using AAV. Our results showed that miR-204 overexpression could alleviate body weight (BW) and heart weight (HW) loss induced by DOX (Figure 3(a)). In addition, we found an increase in the ratio of lung weight (LW) to tibia length (TL) and a decline in the ratio of lung weight (LW) to TL in the DOX group; however, these effects were prominently ameliorated in the DOX-induced miR-204 overexpression group (Figure 3(b)). Moreover, echocardiography experiments were performed to estimate the alterations in cardiac contractile function; these measurements (left ventricular end-diastolic diameter [LVEDd] and LV ejection fraction [EF]) indicated that DOX-treated mice exhibited deteriorated cardiac dysfunction compared with the miR-204 overexpression group (Figure 3(c)). Notable declines in the maximal rate of the increase in left ventricular pressure (+dP/dt) and the maximal rate of the decrease in left ventricular pressure (−dP/dt) were observed in DOX-treated mice, whereas after supplementation with miR-204, significant increases in +dP/dt and −dP/dt were found (Figure 3(d)). The levels of serum biochemical markers (CK-MB, LDH, and cTnI) are effective clinical measures for evaluating cardiac injury; therefore, we examined the serum biochemical levels in DOX-treated mice. We found that serum biochemical levels were higher in the DOX-treated group than in the saline-treated group, while miR-204 overexpression largely ameliorated this elevation (Figure 3(e)).

### 3.4. miR-204 Suppressed Inflammatory Responses and Apoptosis in DOX-Treated Mice

**Figure 4:**
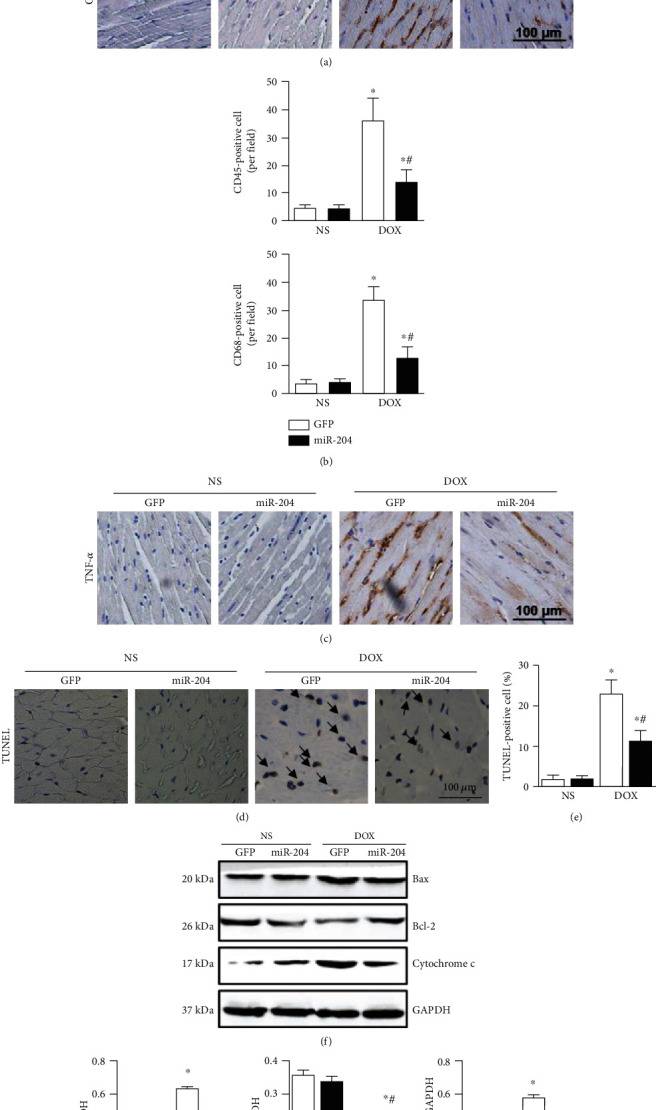
miR-204 inhibited inflammatory responses and apoptosis in mice treated with DOX. (a, b) Representative images and the quantitative analysis of CD45 and CD68 macrophages in the indicated groups (*n* = 4). (c) Representative images of TNF-*α* in each mouse heart (*n* = 4). (d, e) Representative images and quantitative TUNEL results (*n* = 4). (f, g) Western blot and quantitative analysis of apoptosis-related proteins in each group (*n* = 6). ^⁎^*P* < 0.05 versus NS+GFP and ^#^*P* < 0.05 versus DOX+GFP.

As inflammation is induced in DOX-treated mice, we evaluated the effect of miR-204 on inflammation. Our results showed that DOX administration markedly increased the expression of TNF-*α*, the number of CD68-labeled macrophages, and the amount of CD45-labeled leukocyte infiltration in mouse hearts, whereas miR-204 overexpression dramatically attenuated these DOX-induced increases (Figures 4(a)–4(c)). We then examined the severity of cardiomyocyte apoptosis and the potential signaling pathways associated with apoptosis. miR-204 overexpression significantly reduced DOX-induced cardiomyocyte apoptosis, which was confirmed by TUNEL analysis of heart sections (Figures 4(d) and 4(e)).

The expression of Bcl-2 was markedly reduced in the mouse hearts treated with DOX, whereas the expression of Bax and cytochrome c was upregulated in DOX-treated mouse hearts. However, miR-204 overexpression significantly improved the expression of Bcl-2 and attenuated the increased levels of Bax and cleaved cytochrome c induced by DOX (Figures 4(f) and 4(g)).

### 3.5. miR-204 Overexpression Alleviated DOX-Induced Autophagy

**Figure 5:**
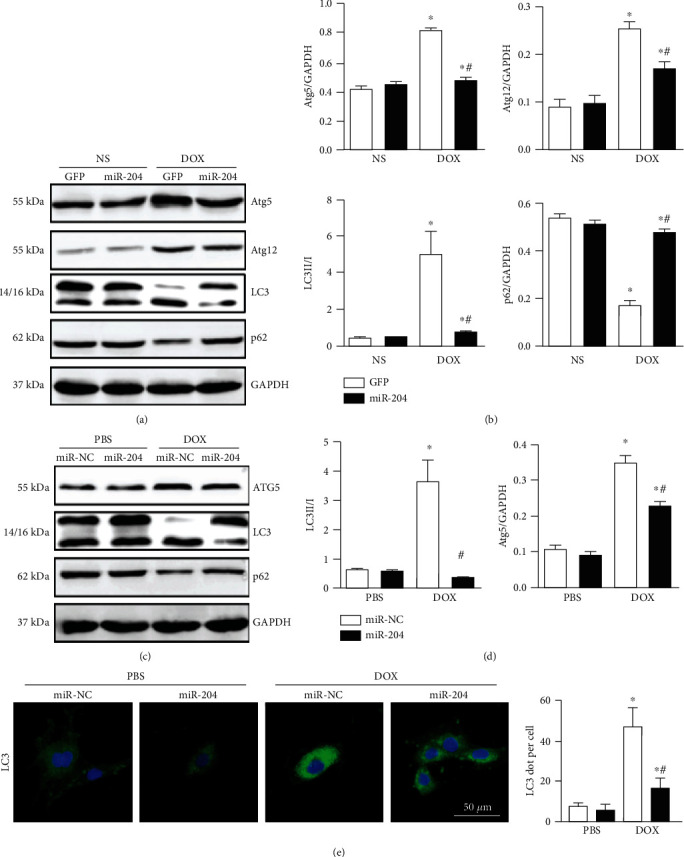
miR-204 supplementation attenuated DOX-induced autophagy in vivo and in vitro. (a, b) The protein levels of ATG5, ATG12, p62, and LC3 in hearts with or without miR-204 (*n* = 6). (c, d) Western blot and quantitative analysis of autophagy-related proteins in each group (*n* = 6). (e) LC3 expression in DOX-treated cardiomyocytes was measured using immunohistochemical analysis (*n* = 4). For (b) ^∗^*P* < 0.05 versus NS+GFP and ^#^*P* < 0.05 versus DOX+GFP. For (d, e), ^∗^*P* < 0.05 versus PBS+miR-NC and ^#^*P* < 0.05 versus DOX+miR-NC.

DOX can cause cardiomyocyte autophagy, which is the main mechanism of DOX-induced cardiotoxicity [19]. Therefore, the effect of miR-204 was evaluated. As shown in Figures 5(a)–5(d), miR-204 overexpression attenuated the decrease in p62 expression induced by DOX in both mouse hearts and cardiomyocytes. In addition, autophagy-related gene expression (Atg5 and Atg12) and the LC3II/I ratio were increased significantly in both DOX-induced hearts and DOX-stimulated cardiomyocytes; however, overexpressing miR-204 reversed these effects. Cardiomyocytes were then subjected to immunofluorescence staining of LC3 (green) to detect the effect of miR-204 on autophagy. Our results showed that DOX treatment increased autophagic vesicle formation in nuclei, whereas the overexpression of miR-204 significantly attenuated the DOX-induced effect (Figure 5(e)).

### 3.6. miR-204 Targets HMGB1 in DOX-Induced Cardiac Injury

**Figure 6:**
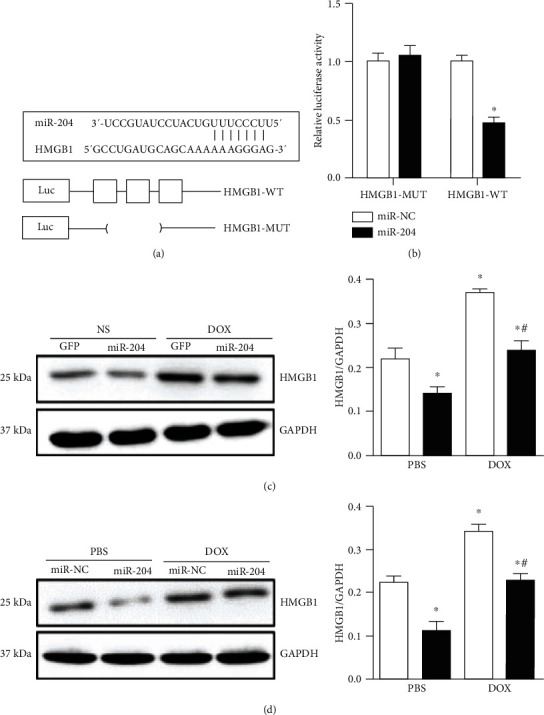
miR-204 targets HMGB1 in DOX-induced cardiac injury. (a) The binding site of miR-204 and HMGB1. (b) The luciferase activity of HMGB1-UTR (HMGB1-WT) or mutant (HMGB1-MUT) in the indicated groups (*n* = 4), ^∗^*P* < 0.05 versus miR-NC. (c) Western blot analysis was performed to measure HMGB1 protein levels in hearts with or without miR-204 (*n* = 6), ^∗^*P* < 0.05 versus NS+GFP and ^#^*P* < 0.05 versus DOX+GFP. (d) Western blots and quantitative analysis of HMGB1 in each group (*n* = 6), ^∗^*P* < 0.05 versus PBS+miR-NC and ^#^*P* < 0.05 versus DOX+miR-NC.

Next, we explored the exact mechanisms by which miR-204 protects against DOX-induced heart injury. Among the putative targets of miR-204, we focused on high mobility group box 1 (HMGB1), which has been reported to regulate cardiac injury induced by DOX [20]. As shown in Figure 5(a), the RNA sequence alignment showed that the 3′-UTR of HMGB1 mRNA contained a complementary site for the seed region of miR-204 (Figure 6(a)). To validate this prediction, wild-type or mutant HMGB1 binding sites in miR-204 were cloned into a luciferase reporter plasmid and then transfected into H9c2 cells. Our results showed that miR-204 overexpression could inhibit the activity of HMGB1-WT, whereas the activity of HMGB1-MUT did not change with the increased expression of miR-204 (Figure 6(b)), indicating the direct interaction of HMGB1 and miR-204. We then assessed whether the HMGB1 protein expression level was altered in cardiomyocytes in vivo and in vitro following DOX treatment. As demonstrated by Western blot analysis, compared with the control group, the DOX-treated group exhibited markedly increased HMGB1 protein expression levels, which were attenuated upon miR-204 overexpression (Figures 6(c) and 6(d)).

### 3.7. HMGB1 Overexpression Abolished the Anti-Inflammatory and Antiapoptotic Effects of miR-204

**Figure 7:**
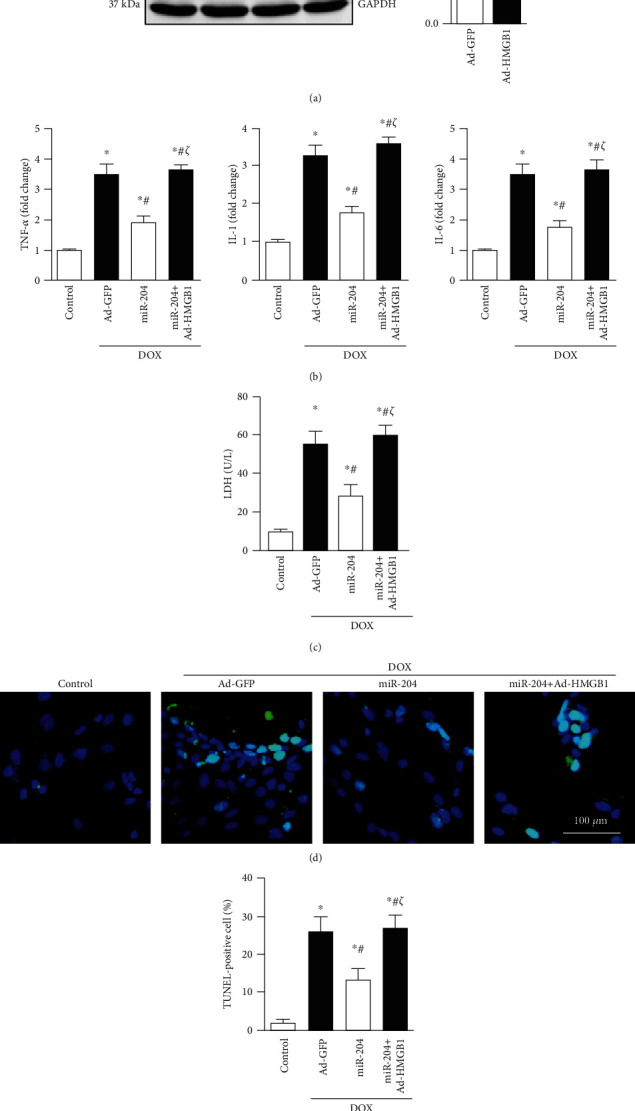
HMGB1 overexpression abolished the anti-inflammatory and antiapoptotic effects of miR-204. (a) The protein expression of HMGB1 after cells were treated with Ad-HMGB1 (*n* = 6), ^∗^*P* < 0.05 vs. Ad-GFP. (b) The mRNA levels of inflammation-related genes (*n* = 6). (c) LDH level in the indicated groups (*n* = 6). (d, e) Representative images and quantitative analysis of TUNEL staining (*n* = 4). ^∗^*P* < 0.05 versus control, ^#^*P* < 0.05 versus DOX+Ad-GFP, and ^*ζ*^*P* < 0.05 versus DOX+miR-204.

To further clarify whether HMGB1 is the target of miR-204 in DOX-induced heart injury, we then determined whether HMGB1 overexpression could abolish the protective effect of miR-204. H9c2 cells were transfected with Ad-HMGB1 to induce overexpression of HMGB1 (Figure 7(a)). After transduction, the mRNA expression levels of TNF-*α*, IL-1, and IL-6 revealed that miR-204 overexpression could inhibit DOX-induced inflammation in vitro, and miR-204 lost its anti-inflammatory effect with HMGB1 overexpression (Figure 7(b)). The TUNEL staining and LDH results showed that miR-204 could alleviate DOX-induced cell loss in vitro, while high expression of HMGB1 almost completely counteracted the antiapoptotic effect of miR-204 (Figures 7(c)–7(e)).

### 3.8. HMGB1 Overexpression Abolished miR-204-Mediated Autophagy Inhibition

**Figure 8:**
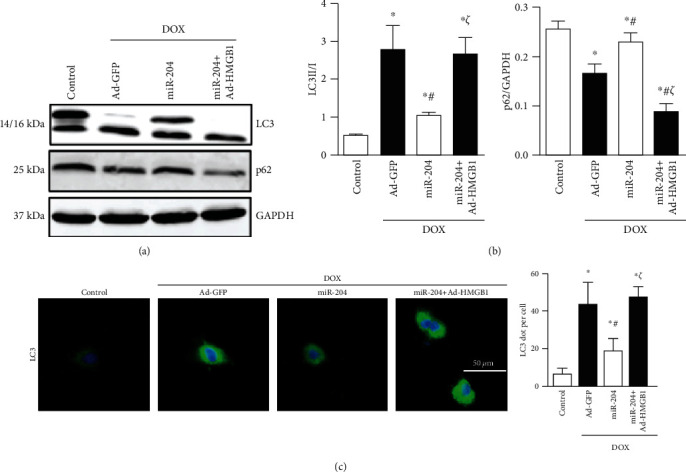
HMGB1 overexpression abolished the antiautophagy effects of miR-204. (a, b) Western blot and quantitative analysis of LC3 and p62 protein expression in each group (*n* = 6). (c) LC3 expression in the indicated groups was measured using immunohistochemical analysis (*n* = 4). ^∗^*P* < 0.05 versus control, ^#^*P* < 0.05 versus DOX+Ad-GFP, and ^*ζ*^*P* < 0.05 versus DOX+miR-204.

To further validate the mechanism, we measured the miR-204-mediated autophagy inhibition after treatment with Ad-HMGB1. The results showed that overexpressing miR-204 ameliorated the decrease in p62 levels and the increase in the LC3II/I ratio induced by DOX, while overexpressing HMGB1 almost completely offset the effects of miR-204 (Figures 8(a) and 8(b)). Immunofluorescence staining of LC3 further demonstrated that HMGB1 overexpression almost completely counteracted miR-204-mediated autophagy inhibition in H9c2 cells (Figure 8(c)).

## 4. Discussion

In this study, we examined the effect of miR-204 on DOX-induced myocardial damage in a murine cardiotoxicity model. We found that overexpression of miR-204 could reduce DOX-induced myocardial damage and improve cardiac function in mice. We further found that miR-204 protected against myocardial injury by regulating the HMGB1 pathway. Moreover, we found that the protective effect of miR-204 supplementation was counteracted by the overexpression of HMGB1 in mice. In summary, our study showed that miR-204 supplementation could provide a protective effect against DOX-induced cardiotoxicity by inhibiting the HMGB1 pathway.

In the past few decades, there has been growing interest in studying the roles of miRNAs in cardiovascular diseases. Many miRNAs, including miR-21, miR-29b, and miR-378, are involved in DOX-induced cardiac injury [16, 21, 22]. The role of miR-204 in many diseases has been extensively studied; however, it is still not known whether miR-204 is involved in DOX-induced cardiac injury. First, the present study showed that the expression level of miR-204 was decreased in DOX-treated hearts, and the alteration in miR-204 expression after DOX treatment suggested that miR-204 might play a vital role in DOX-induced cardiac injury. DOX-induced cardiotoxicity is characterized by increased activities of serum biomarkers of myocardial toxicity, body weight loss, and left ventricular dysfunction [17–19]. This study suggested that DOX administration could cause severe left ventricular dysfunction. In addition, the increase in serum biomarkers of myocardial toxicity indicates the loss of cardiomyocyte membrane integrity, and these results are consistent with the findings of previous studies. Interestingly, we found that overexpression of miR-204 ameliorated all DOX-induced myocardial toxicities, as evidenced by the amelioration of cardiac damage and dysfunction and the lowering of cardiotoxic biomarkers.

Previous studies have indicated that myocardial inflammatory cytokines were activated in DOX-induced cardiotoxicity and cardiac function could be improved with the alleviation of inflammatory cytokines [17, 23]. In the present study, DOX-induced myocardial toxicity induced an apparent increase in proinflammatory cytokines; however, miR-204 overexpression significantly alleviated these inflammatory responses. Our results seem to be consistent with those of Li et al., who reported that miR-204 played a protective role in human trabecular meshwork cells by inhibiting inflammation [24].

There is increasing evidence that programmed cell death, including apoptosis and autophagy, is an important cause of DOX-induced heart injury [12, 25]. Therefore, the inhibition of DOX-induced apoptosis and autophagy may be a new therapeutic strategy to reduce DOX-induced heart injury. In our study, we found that the expression of Bax and cytochrome c was reduced, and the protein expression of Bcl-2 was increased after DOX treatment. However, miR-204 overexpression restored the changes in cytochrome c activity, Bax, and Bcl-2 induced by DOX. Studies have shown that the inhibition of autophagy could attenuate DOX-induced cardiotoxicity [26]. In the present study, we found that the expression of LC3, ATG5, and ATG12 was increased in response to DOX treatment but was effectively suppressed by miR-204 overexpression. These results showed that miR-204 overexpression exerted inhibitory effects on DOX-induced apoptosis and autophagy.

Autophagy is a self-degradative process. A low level of constitutive autophagy has an important role in the normal turnover of proteins and organelles. It seems that autophagy is a cytoprotective mechanism against cell death. Nevertheless, autophagy might also become harmful and promote cell death under specific conditions [27]. The role of autophagy in DOX-induced myocardial injury has been widely debated. Zilinyi reported that autophagy was impaired in DOX-induced cardiac injury, and restoring autophagy could increase cardiomyocyte survival [28]. Ye et al. showed that DOX-induced cardiac injury was associated with increased autophagosome levels by transmission electron micrographs [29]. In this study, we found that the autophagy level was increased in both mouse hearts and H9c2 cells treated with DOX and that miR-204 exerts a protective effect by inhibiting autophagy.

It is well known that miRNAs exert their biological functions by binding with the 3′-UTR of target genes [21]. In our study, we confirmed that miR-204 directly binds to the 3′-UTR of HMGB1. HMGB1 is a DNA-bound nuclear protein that is stimulated and passively released by cytokine stimulation during cell injury and death. A previous study showed that HMGB1 plays vital roles in the development of cardiovascular diseases [30]. Yao et al. found that HMGB1 was involved in DOX-induced cardiomyocyte apoptosis and cardiac insufficiency, and inhibiting HMGB1 could alleviate DOX-induced cardiomyopathy [20]. In addition, HMGB1 could directly interact with Beclin1 and ATG5 and block their calpain-mediated cleavage during inflammation, thereby maintaining proautophagic functions [27]. In this study, we demonstrated that the expression of HMGB1 was increased after DOX treatment, while the level of HMGB1 was reduced with miR-204 supplementation in vivo and in vitro. In addition, rescue experiments showed that HMGB1 supplementation abolished the protective effect of miR-204 on DOX-induced cardiac injury, indicating that miR-204 partially exerts myocardial protection by regulating the HMGB1 pathway. Li et al. reported that plasma miR-204 expression in children with congenital heart disease with pulmonary hypertension was negatively correlated with the degree of pulmonary hypertension and that miR-204 expression may be an indicator of pulmonary hypertension severity [31]. This result is inconsistent with our results that miR-204 acted as a protective factor during DOX-induced cardiac injury. The different diseases and the underlying mechanisms that cause pulmonary hypertension (persistent overload) may be responsible for these inconsistent results.

## 5. Conclusions

In conclusion, this study confirmed that miR-204 improved cardiac function and inhibited inflammation, apoptosis, and autophagy in cardiomyocytes partly through regulating the HMGB1 pathway in a DOX-induced cardiotoxicity model. We believe that the newly discovered miR-204/HMGB1 axis may provide a research strategy for studying the potential molecular mechanism of cardiotoxicity induced by chemotherapeutic drugs.

## Data Availability

The data used to support the findings of this study are included within the article.
